# New records for the *Helius* Lepeletier & Serville fauna (Diptera, Limoniidae) of China

**DOI:** 10.3897/BDJ.12.e115775

**Published:** 2024-05-31

**Authors:** Hanhuiying Lv, Yuanyuan Xu, Yufei Zhao, Kejian Lin, Xiao Zhang

**Affiliations:** 1 Key Laboratory of Biohazard Monitoring and Green Prevention and Control in Artificial Grassland, Ministry of Agriculture and Rural Affairs, Institute of Grassland Research, Chinese Academy of Agricultural Sciences, Hohhot 010010, China Key Laboratory of Biohazard Monitoring and Green Prevention and Control in Artificial Grassland, Ministry of Agriculture and Rural Affairs, Institute of Grassland Research, Chinese Academy of Agricultural Sciences Hohhot 010010 China; 2 Shandong Engineering Research Center for Environment-Friendly Agricultural Pest Management, College of Plant Health and Medicine, Qingdao Agricultural University, Qingdao 266109, China Shandong Engineering Research Center for Environment-Friendly Agricultural Pest Management, College of Plant Health and Medicine, Qingdao Agricultural University Qingdao 266109 China

**Keywords:** Chinese fauna, crane fly, Elephantomyiini, Limoniinae, new geographical record, taxonomy

## Abstract

**Background:**

The genus *Helius* Lepeletier & Serville, 1828 is globally distributed with 232 species and subspecies, of which 25 have been known to occur in China. Amongst the Chinese *Helius* crane flies, 24 species are distributed in southern China. The species diversity of *Helius* in other Chinese regions may be severely underestimated due to a lack of investigation. Some investigations on crane flies in Inner Mongolia, China have been initiated by the authors together with other entomologists, with *Helius* being one of the key targets of attention.

**New information:**

Two *Helius* species, H. (Helius) flavus (Walker, 1856) and H. (H.) gracillimus Alexander, 1938, are added to the Chinese fauna. The two newly-recorded species also represent the first records of the crane fly tribe Elephantomyiini in Inner Mongolia. Re-descriptions and illustrations of the two newly-recorded species are presented.

## Introduction

*Helius* Lepeletier & Serville, 1828 (Latreille et al. 1828) is a species-rich genus in the family Limoniidae consisting of extant 232 species and subspecies from nine subgenera around the world (Oosterbroek 2023). This genus and another limoniid genus *Elephantomyia* Osten Sacken, 1860 (Osten Sacken 1860), both with elongated rostrum, are often considered as a sister group (such as Ribeiro (2008), Petersen et al. (2010), Kang et al. (2023)). It is worth mentioning that there are many records of *Elephantomyia* feeding on flowers (such as Alexander (1924a), Savchenko (1983), Savchenko (1986), Zhang et al. (2015), Kato (2017)), but there seems to be no published record of *Helius* feeding on flowers or of feeding in any other way (Oosterbroek and Lukashevich 2021).

In the past three decades, a large number of taxonomic studies have been carried out on the genus *Helius*, mainly focusing on fossil species (such as Krzemiński (1991), Krzemiński (1993), Krzemiński (2002), Podenas (2002), Ribeiro (2003), Kania et al. (2013), Kania (2014), Krzemiński et al. (2014), Kania et al. (2016a), Kania et al. (2016b), Kopeć et al. (2016), Kania et al. (2018), Wu et al. (2019), Kania-Klosok et al. (2021)), with relatively few on extant species. Stubbs (1992) reported a *Helius* species new to Britain. Welch and Gelhaus (1994) published a new *Helius* species from Trinidad. Podenas and Byun (2014), Podenas et al. (2015) and Podenas et al. (2017) reported five newly-recorded species of *Helius* from the Korean Peninsula and provided their descriptions and illustrations. Quindroit (2022) provides biologies, geographical distribution data and illustrations of five *Helius* species from France. Xu et al. (2023) reported the genus *Helius* in Guangxi, China for the first time and published a new species and a newly-recorded subspecies from China.

According to Oosterbroek (2023), 25 *Helius* crane fly species have been recorded from China, of which 23 belong to the nominotypical subgenus and two belong to the subgenus Eurhamphidia Alexander, 1915 (Alexander 1915). In this study, we report two additional *Helius* species to the Chinese fauna, namely H. (Helius) flavus (Walker, 1856) and H. (H.) gracillimus Alexander, 1938, which also represent the first records of the crane fly tribe Elephantomyiini in Inner Mongolia, China. Re-descriptions and illustrations of the newly-recorded species are presented.

## Materials and methods

**Figure 1.**
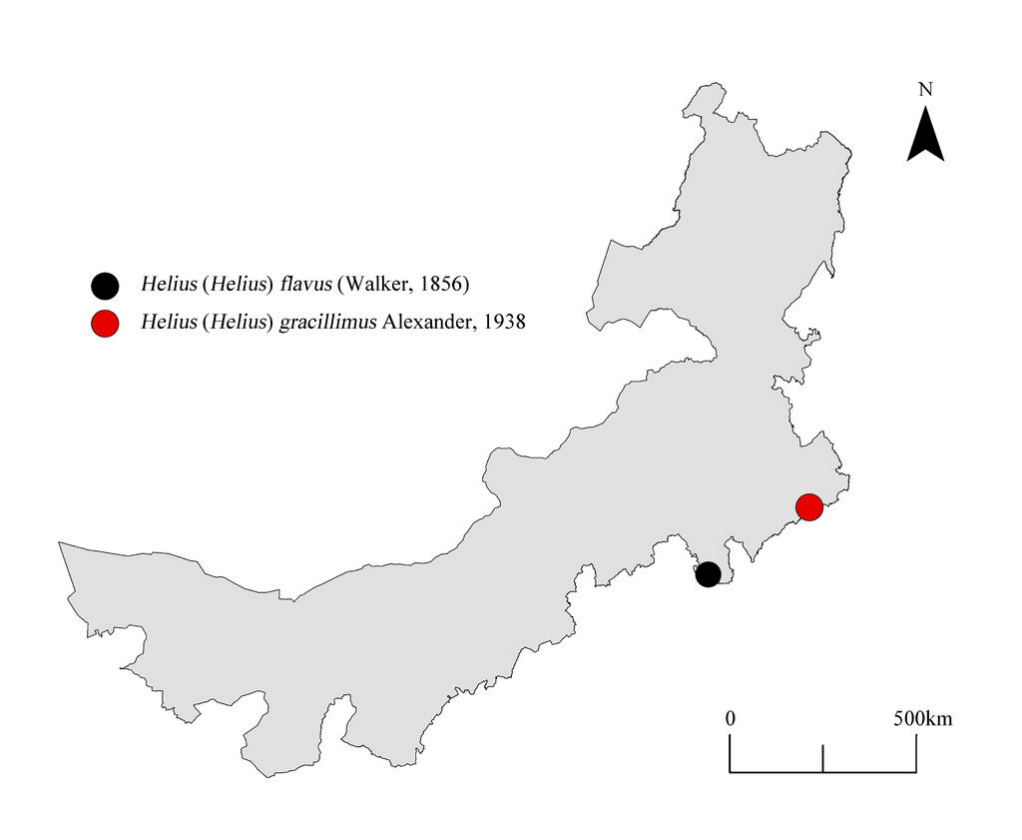
The distribution map of *Helius* crane flies in Inner Mongolia, China.

The specimens of this study were collected by insect nets at different locations in Inner Mongolia, China (Fig. 1) and deposited in the Entomological Museum of Qingdao Agricultural University, Shandong, China (QAU). Genitalic preparations of males were made by boiling the hypopygium in lactic acid (C_3_H_6_O_3_) for half an hour. Details of body colouration were examined in specimens immersed in 75% ethanol (C_2_H_5_OH). Specimens were examined using a ZEISS Stemi 2000-C stereomicroscope. Photographs were captured by a Canon EOS 5D Mark IV digital camera.

The morphological terminology mainly follows Cumming and Wood (2017) and that for venation follows de Jong (2017). The following abbreviations in figures are used: aa = aedeagus apodeme; aed = aedeagus, cerc = cercus, goncx = gonocoxite, hyp vlv = hypogynial valve, i gonst = inner gonostylus, interb = interbase; o gonst = outer gonostylus, pm = paramere, st = sternite, tg = tergite.

## Taxon treatments

### Helius (Helius) flavus

(Walker, 1856)

4ADCF6EC-91A9-5676-9E16-DDE3C56F42A3

Rhamphidia flava Walker, 1856 - Walker (1856): 309.
Helius flavus (Walker, 1856) - Starý (1966): 86.
Helius (Helius) flavus (Walker, 1856) - Podenas et al. (2017): 272.

#### Materials

- **Type status:** Other material. **Occurrence:** recordedBy: Xingyang Qian; individualCount: 4; sex: 2 males, 2 females; lifeStage: adult; occurrenceID: 95CD7DBB-9F0D-5B03-A9E5-7B85ACC3ADE7; **Taxon:** scientificName: Helius (Helius) flavus (Walker, 1856); order: Diptera; family: Limoniidae; genus: Helius; subgenus: Helius; **Location:** country: China; stateProvince: Inner Mongolia; county: Ningcheng; locality: Heilihe National Nature Reserve, Xidaliang; verbatimElevation: 1070m; decimalLatitude: 41.399511; decimalLongitude: 118.361483; **Event:** samplingProtocol: sweeping; eventDate: 30-Jul-22; **Record Level:** institutionCode: QAU

#### Description

**Diagnosis.** Antenna with basal flagellomeres oval. Rostrum about 1.2 times as long as remainder of head. Prescutum and presutural scutum yellow, darker at anterior third. Femora brownish-yellow, darkened at tip. Wing with apex slightly darkened, stigma inconspicuous. Venation with Sc ending opposite about 3/4 of Rs, cell dm about twice as long as wide, m-cu beyond fork of M. Posterior margin of tergite 9 with a broad and deep V-shaped notch. Outer gonostylus with tip black and bifid. Inner gonostylus expanded near middle and bent inwards at a right angle, distal part slender; outer margin of expanded lobe with five teeth. Interbase distally horn-shaped, tip suddenly thinning into a spine. Aedeagus rod-shaped, tip slightly expanded and rounded.

**Figure 2.**
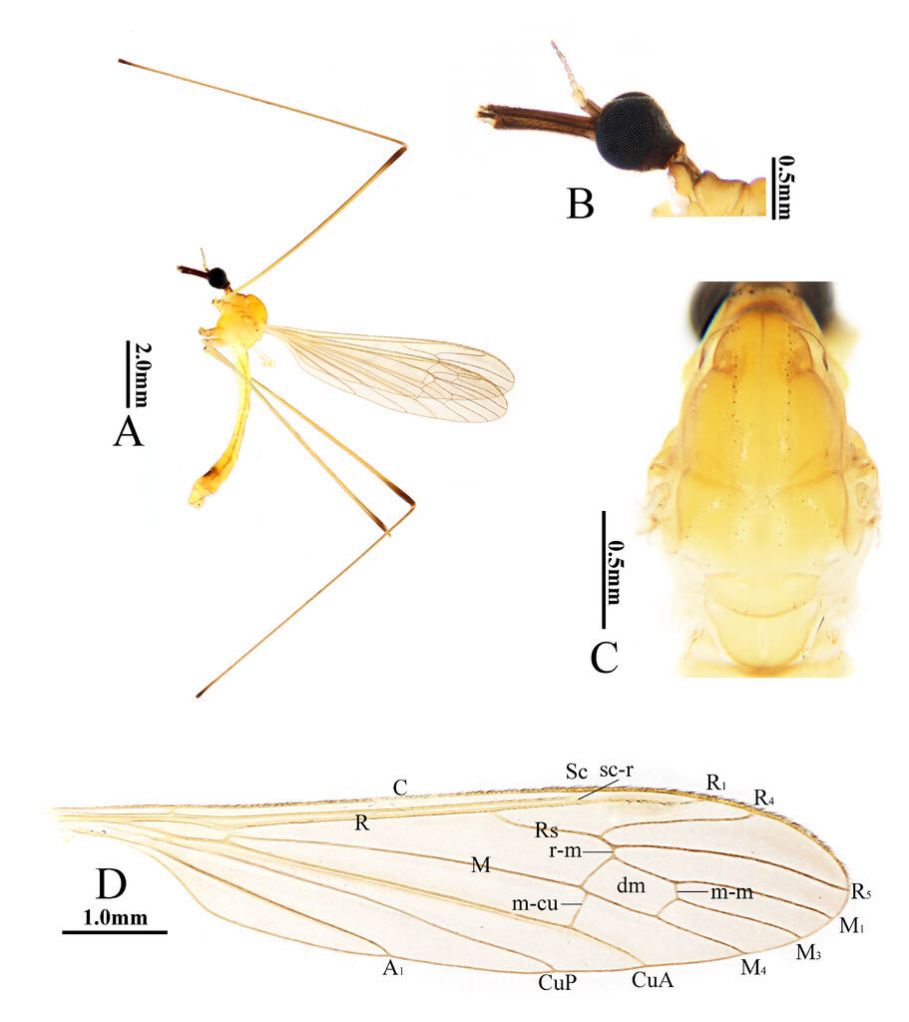
Helius (Helius) flavus (Walker, 1856). **A** habitus of male, lateral view; **B** head, lateral view; **C** thorax, dorsal view; **D** wing. Scale bars: 2.0 mm (A); 0.5 mm (B, C); 1.0 mm (D).

**Male** (Fig. 2A). Body length 7.1–7.3 mm (excluding rostrum), wing length 7.5–7.7 mm, rostrum length 0.9–1.0 mm, halter length 1.1–1.3 mm.

Head (Fig. 2B). Brownish-black. Setae on head dark brown. Antenna with scape dark brown, pedicel yellow to pale brownish-yellow, flagellomeres brown with basal segments yellow to pale brownish-yellow. Scape cylindrical, 2.5 times as long as wide. Pedicel oval, tip slightly enlarged. Flagellomeres 1–3 oval; remaining flagellomeres cylindrical, tapering apically and elongated, with dark brown verticils. Rostrum about 1.2 times as long as remainder of head, dark brown with brownish-black setae. Palpus dark brown with brownish-black setae.

Thorax (Fig. 2C). Pronotum dirty yellow with middle area brown. Prescutum and presutural scutum yellow, darker at anterior third, dorsally a pair of yellow stripes bordered by brown dots and separated by narrow pale line. Postsutural scutum pale yellow, each lobe with oval area bordered by dark spots. Scutellum and mediotergite yellow. Pleuron yellow to brownish-yellow (Fig. 2A). Setae on thorax dark brown. Coxae brownish-yellow; trochanters brownish-yellow with fore trochanter slightly paler; femora brownish-yellow, darkened at tips; tibiae pale brown with tips dark brown. Setae on legs brown. Wing (Fig. 2D) pale brown with base and costal area yellow, wing apex slightly darkened; stigma inconspicuous. Veins brown. Venation: Sc ending opposite about 3/4 of Rs, sc-r near tip of Sc; m-m shorter than basal section of M_3_; cell dm about twice as long as wide; m-cu about 1/3 of its length beyond fork of M. Halter pale yellow.

Abdomen. Tergites yellow to brownish-yellow. Sternites 1–6 yellow, sternite 7 brownish-yellow with caudal third brown, sternite 8 brownish-yellow with a brown spot at middle, sternite 9 brownish-yellow. Setae on abdomen brown.

**Figure 3.**
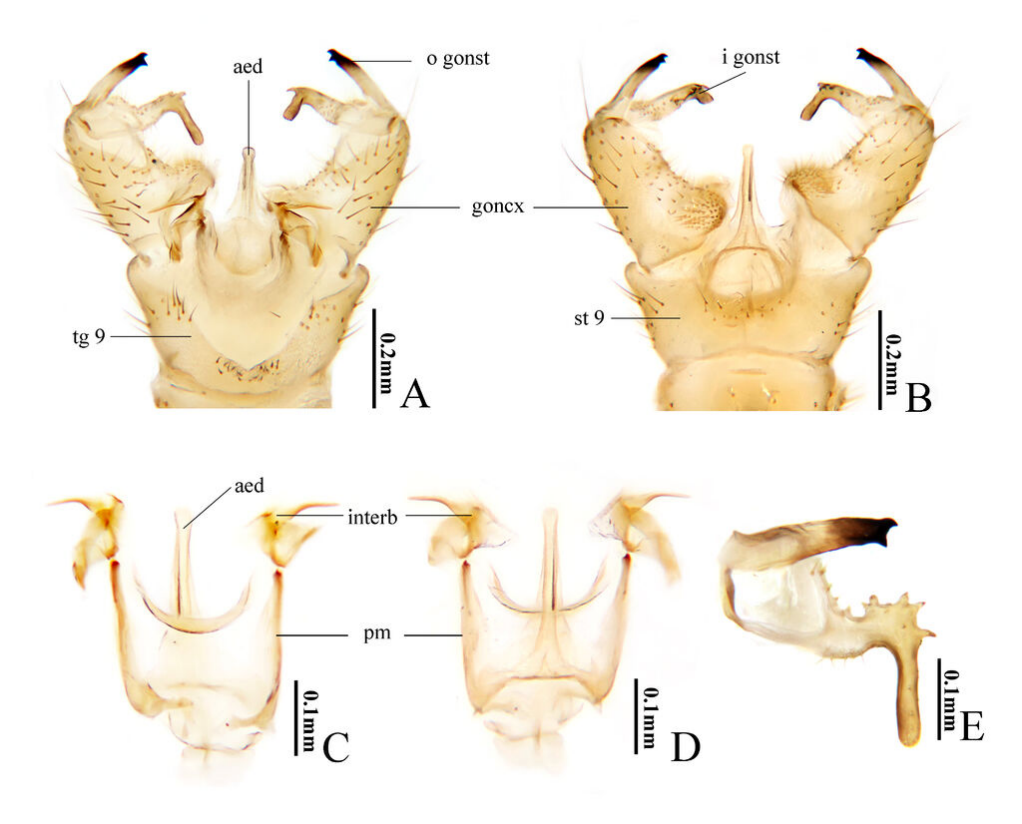
Helius (Helius) flavus (Walker, 1856). **A** male hypopygium, dorsal view; **B** male hypopygium, ventral view; **C** aedeagal complex, dorsal view; **D** aedeagal complex, ventral view; **E** outer gonostylus and inner gonostylus, dorsal view. Scale bars: 0.2 mm (A, B); 0.1 mm (C, D, E).

Hypopygium (Fig. 3). Generally yellow. Posterior margin of tergite 9 with a broad and deep V-shaped notch (Fig. 3A). Gonocoxite cylindrical, tip round, base with a stout spinerous lobe (Fig. 3A and B). Outer gonostylus nearly straight; tip black and bifid, outer spine slightly bent, inner spine triangle (Fig. 3A, B and E). Inner gonostylus expanded near middle and bent inwards at a right angle, distal part slender; outer margin of expanded lobe with five teeth (Fig. 3A, B and E). Interbase distally horn-shaped, tip suddenly thinning into a spine; base horizontally extended (Fig. 3A, C and D). Parameres sheet-like, medially fused, apically elongated and connecting to base of interbase (Fig. 3C and D). Aedeagus rod-shaped, tip slightly expanded and rounded, base expanded (Fig. 3A–D).

**Figure 4.**
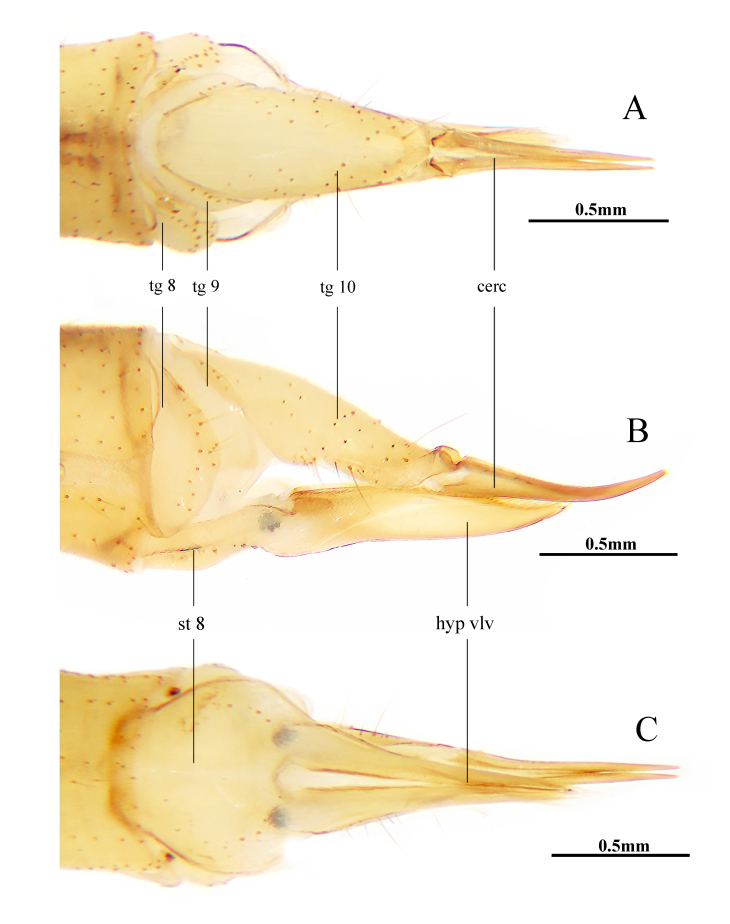
Helius (Helius) flavus (Walker, 1856). **A** female ovipositor, dorsal view; **B** female ovipositor, lateral view; **C** female ovipositor, ventral view. Scale bars: 0.5 mm (A, B, C).

**Female** (Fig. 4). Body length 9.6–10.2 mm (excluding rostrum), wing length 8.7–9.2 mm, rostrum length 1.1–1.2 mm, halter length 1.3–1.4 mm. Generally similar to male by body colouration, except abdomen with segments 7 and 8 yellow. Ovipositor with tergites 9 and 10 yellow (Fig. 4A and B). Cercus brownish-yellow, slightly darker at subtip, tip raised and acute (Fig. 4A and B). Hypogynial valve yellow to brownish-yellow, nearly straight, tip reaching slightly beyond middle of cercus (Fig. 4B and C).

#### Distribution

China (Inner Mongolia); Austria, Belarus, Belgium, Bulgaria, Czech Rep., Denmark, Finland, France, Germany, Great Britain, Hungary, Ireland, Italy, Kazakhstan, Lithuania, Malta, Netherlands, North Caucasus, North Macedonia, Norway, Poland, Romania, Russia, Serbia, Slovakia, South Korea, Sweden, Switzerland, Ukraine (Oosterbroek 2023).

#### Notes

Helius (H.) flavus is widely distributed in the Palaearctic Region (Oosterbroek 2023) and recorded in China for the first time. Walker (1856) first discovered this species and provided a brief description without figures and Starý (1966) illustrated the female ovipositor for the first time. Subsequently, Starý and Rozkośný (1970), Geiger (1986), Podenas et al. (2006), Podenas et al. (2017) and Quindroit (2022) successively illustrated this species, amongst which Podenas et al. (2017) provided detailed description and illustrations of the male for this species. The species is closely related to H. (H.) unicolor (Brunetti, 1912) in the key by Xu et al. (2023) and can be distinguished by the inner gonostylus with five teeth on the outer margin of the middle (Fig. 3A, B and E). In H. (H.) unicolor, the inner gonostylus has no teeth (Brunetti 1912).

### Helius (Helius) gracillimus

Alexander, 1938

93159B9A-B621-5022-A7CE-9E791A64FFCA

Helius (Helius) gracillimus Alexander, 1938 - Alexander (1938): 143.

#### Materials

- **Type status:** Other material. **Occurrence:** recordedBy: Yan Li; individualCount: 3; sex: males; lifeStage: adult; occurrenceID: 4932F87B-166F-50C2-AD8F-FB35D8AB10CA; **Taxon:** scientificName: Helius (Helius) gracillimus Alexander, 1938; order: Diptera; family: Limoniidae; genus: Helius; subgenus: Helius; **Location:** country: China; stateProvince: Inner Mongolia; county: Horqin Left Back Banner; locality: Daqinggou National Nature Reserve; decimalLatitude: 42.76881; decimalLongitude: 122.21064; **Event:** samplingProtocol: sweeping; eventDate: 21-Aug-14; **Record Level:** institutionCode: QAU

#### Description

**Diagnosis.** Antenna long with base of first flagellomere paler, flagellomeres long cylindrical. Rostrum slightly longer than remainder of head. Prescutum and presutural scutum pale yellow dorsally with a pair pale yellow stripes separated by a narrow line and bordered by dark dots. Femora and tibiae pale brownish-yellow with tips slightly darker. Wing with large and brown stigma, brown spot at base of Rs, brown seam along cord. Venation with Sc ending nearly at fork of Rs, cell dm about 2.5 times as long as wide, m-cu beyond fork of M. Posterior margin of tergite 9 arched medially. Outer gonostylus slender and slightly curved with blackened and obtuse tip. Inner gonostylus with distal half curved and tapering apically. Interbase long, blade-shaped, outer margin darker. Aedeagus rod-shaped, tip expanded and trifid, middle spine tube-shaped and wavy, lateral spines triangle and shorter.

**Figure 5.**
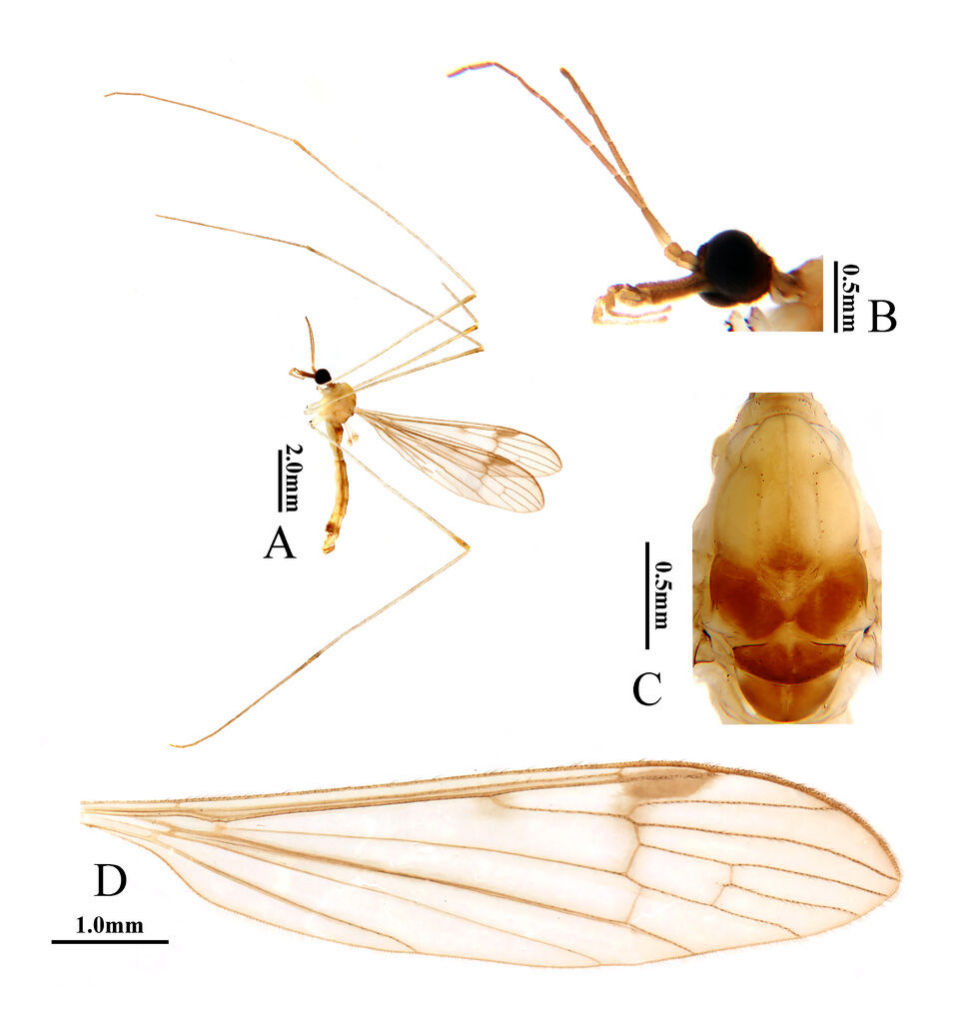
Helius (Helius) gracillimus Alexander, 1938. **A** habitus of male, lateral view; **B** head, lateral view; **C** thorax, dorsal view; **D** wing. Scale bars: 2.0 mm (A); 0.5 mm (B, C); 1.0 mm (D).

**Male** (Fig. 5A). Body length 6.1–6.7 mm (excluding rostrum), wing length 7.0–7.8 mm, rostrum length 0.5–0.6 mm, halter length 1.0–1.2 mm.

Head (Fig. 5B). Brownish-black. Setae on head brown. Antenna long, brown with base of first flagellomere paler. Scape cylindrical, twice as long as wide. Pedicel oval, tip slightly enlarged. Flagellomeres long cylindrical, tapering apically, with brown verticils. Rostrum slightly longer than remainder of head, dark brown with brownish-black setae. Palpus brown to dark brown with brownish-black setae, terminal segment elongated.

Thorax (Fig. 5C). Pronotum yellow with middle area slightly darker. Prescutum and presutural scutum pale yellow, dorsally with a pair pale yellow stripes separated by a narrow line and bordered by dark dots; base of lines darkened by brown. Postsutural scutum brown, each lobe with a spot indistinctly bordered by pale yellow. Scutellum brown to dark brown. Mediotergite brown with indistinct median line. Pleuron brownish-yellow (Fig. 5A). Setae on thorax dark brown. Coxae yellow with fore coxa slightly darker, trochanters yellow, femora and tibiae pale brownish-yellow with tips slightly darker, tarsi brown to dark brown. Setae on legs brown. Wing (Fig. 5D) pale brown with cell sc darker; stigma large and dark brown, brown spot at base of Rs, brown seam along cord. Veins brown. Venation: Sc ending nearly at fork of Rs, sc-r near tip of Sc; m-m shorter than basal section of M_3_; cell dm about 2.5 times as long as wide; m-cu more than 1/2 of its length beyond fork of M. Halter brownish-yellow with knob darker.

Abdomen. Tergites 1–8 brown, yellow laterally, with margins darker; tergite 9 brown. Sternites 1–8 yellow to brownish-yellow, sternite 9 dark brown. Setae on abdomen brown.

**Figure 6.**
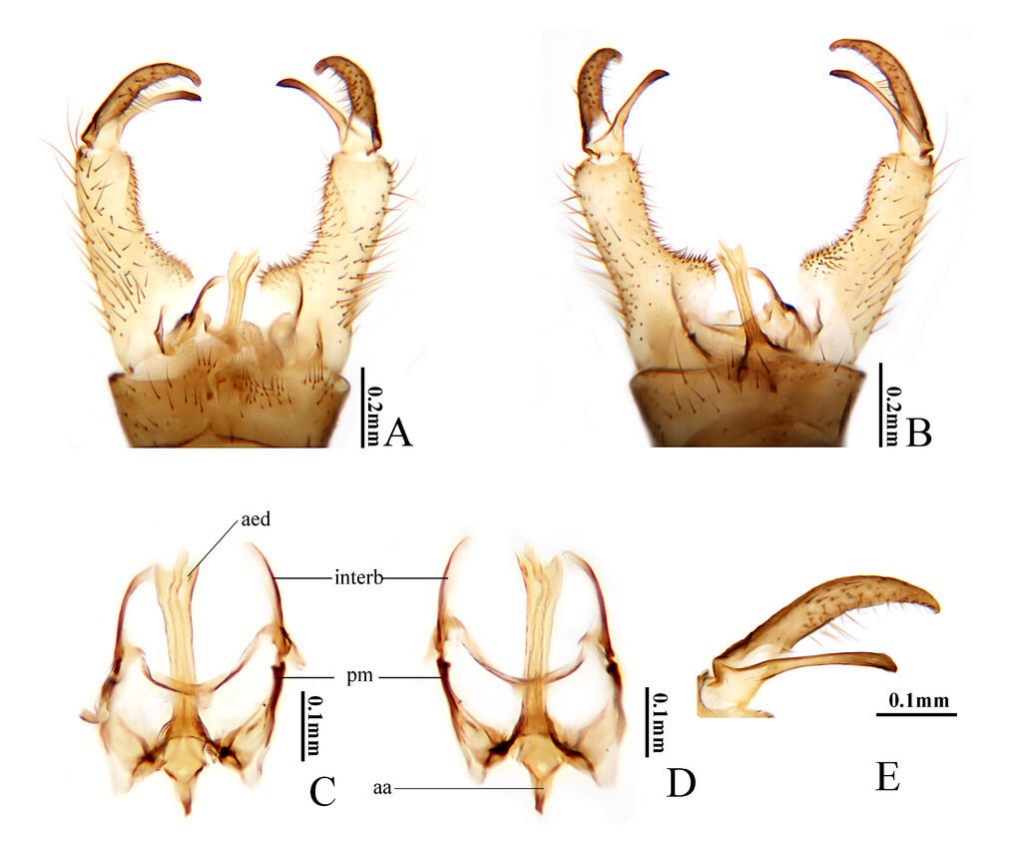
Helius (Helius) gracillimus Alexander, 1938. **A** male hypopygium, dorsal view; **B** male hypopygium, ventral view; **C** aedeagal complex, dorsal view; **D** aedeagal complex, ventral view; **E** outer gonostylus and inner gonostylus, dorsal view. Scale bars: 0.2 mm (A, B); 0.1 mm (C, D, E).

Hypopygium (Fig. 6). Posterior margin of tergite 9 arched medially (Fig. 6A). Gonocoxite yellow, cylindrical, base with a stout, spinerous lobe (Fig. 6A and B). Outer gonostylus brownish-yellow, slender and slightly curved, tip blackened and obtuse (Fig. 6A, B and E). Outer gonostylus about 2/3 as long as inner gonostylus (Fig. 6E). Inner gonostylus dark brown, distal half curved and tapering apically (Fig. 6A, B and E). Interbase long, blade-shaped, outer margin darker (Fig. 6A–D). Parameres sheet-like, medially fused, apically elongated and connecting to base of interbase (Fig. 6C and D). Aedeagus rod-shaped, tip expanded and trifid, middle spine tube-shaped and wavy, lateral spines triangle and shorter (Fig. 6A–D).

**Female.** Unknown.

#### Distribution

China (Inner Mongolia); North Korea, Russia, South Korea (Oosterbroek 2023).

#### Notes

Helius (H.) gracillimus is an East Palaearctic species that occurs in Russia, North Korea and South Korea (Oosterbroek 2023) and now recorded in China for the first time. For descriptions and illustrations of this species, also see Alexander (1938) and Podenas and Byun (2014). The species is closely related to H. (H.) subfasciatus Alexander, 1924 in the key by Xu et al. (2023) and can be distinguished by the brownish-yellow pleuron and the yellow coxae (Fig. 5A). In H. (H.) subfasciatus, the pleuron is dark brownish-black and the coxae is brown (Alexander 1924b).

## Discussion

The two newly-recorded species for Chinese fauna are both from Inner Mongolia, a Chinese Province with very limited species records of Limoniidae. According to Oosterbroek (2023), only five limoniid crane flies are recorded in Inner Mongolia, of which four belong to the subfamily Chioneinae and only one belongs to the subfamily Limoniinae. With the discovery of these two *Helius* species in this study, the number of Limoniinae species in Inner Mongolia increases to three. They also represent a new recorded crane fly tribe (i.e. Elephantomyiini) in Inner Mongolia, which indicates that the species diversity of Limoniidae in Inner Mongolia may be severely underestimated. As for Chinese *Helius* crane flies, previously only one species was distributed in northern China (Jilin), while the remaining 24 species were all distributed in southern China (Oosterbroek 2023). The discovery of the two *Helius* species in Inner Mongolia provides valuable distribution records for geographical research of the genus and also indicates that there may be huge potential for species diversity of *Helius* in northern China.

## Supplementary Material

XML Treatment for Helius (Helius) flavus

XML Treatment for Helius (Helius) gracillimus
